# Differences in labour market marginalisation among young immigrant groups and Swedish-born youth

**DOI:** 10.1093/eurpub/ckac130.079

**Published:** 2022-10-25

**Authors:** R Amin, G Geirsdottir, E Mittendorfer-Rutz, E Björkenstam, L Chen, T Dorner

**Affiliations:** 1 Department of Clinical Neuroscience, Karolinska Institutet, Stockholm, Sweden; 2 Karl-Landsteiner Institute, Health Promotion Research, Vienna, Austria

## Abstract

**Background:**

There is a knowledge gap regarding the risk for labour market marginalisation among younger cohorts of refugees and non-refugee immigrants. We investigated if the risk of long-term unemployment (LTU) and disability pension (DP) differs between young refugees and non-refuge immigrants compared to the Swedish-born. The role of age at arrival, duration of residency and morbidity in this association was also investigated.

**Methods:**

All 19- to 25-year-olds residing in Sweden on 31 December 2004 (1691 refugees who were unaccompanied by a parent at arrival, 24,697 accompanied refugees, 18,762 non-refugee immigrants and 621,455 Swedish-born individuals) were followed from 2005 to 2016 regarding LTU (>180 days annually) and DP using nationwide register data. Cox regression models were used to estimate crude and multivariate-adjusted (adjusted for several socio-demographic, labour market and health-related covariates) hazard ratios (aHRs) with 95% confidence intervals.

**Results:**

Compared to the Swedish-born, all migrant groups had around a 1.8-fold higher risk of LTU (range aHR=1.71-1.83) and around a 30% lower risk of DP (range aHR=0.66-0.76). Older age at arrival was associated with a higher risk of LTU only for non-refugee immigrants. Both older age at arrival and a shorter duration of residency were associated with a lower risk of DP for all migrant groups. Psychiatric morbidity had the strongest effect on subsequent DP, with no significant differences between migrant groups and the Swedish-born (range aHR=5.1-6.1).

**Conclusions:**

Young immigrants had a higher risk of LTU and a lower risk of DP than their Swedish-born peers. No differences between the different immigrant groups were found. Age at arrival, psychiatric morbidity and duration of residency are strong determinants of being granted DP.

**Key messages:**

- Young refugees and non-refugee immigrants both had a similar higher risk of long-term unemployment and a similar lower risk of disability pension than their Swedish-born peers.
- Age at arrival, psychiatric morbidity and duration of residency are strong determinants of being granted disability pension for young refugees and non-refugee immigrants in Sweden.

